# Right Atrial Pressure/Pulmonary Capillary Wedge Pressure Ratio Predicts In-Hospital Mortality in Left Ventricular Assist Device Recipients

**DOI:** 10.3390/jcm14134784

**Published:** 2025-07-07

**Authors:** Berhan Keskin, Aykun Hakgor, Bilge Yilmaz, Korhan Erkanli, Beytullah Cakal, Arzu Yazar, Yahya Yildiz, Bilal Boztosun, Ibrahim Oguz Karaca

**Affiliations:** 1 Department of Cardiology, Medipol Mega University Hospital, Istanbul Medipol University, 34214 Istanbul, Türkiye; 2 Department of Cardiovascular Surgery, Medipol Mega University Hospital, Istanbul Medipol University, 34214 Istanbul, Türkiye; 3 Department of Anesthesiology, Medipol Mega University Hospital, Istanbul Medipol University, 34214 Istanbul, Türkiye

**Keywords:** left ventricular assist device, in-hospital mortality, right atrial pressure, pulmonary capillary wedge pressure

## Abstract

**Background/Objectives**: Right ventricular failure (RVF) is a major contributor to early mortality after left ventricular assist device (LVAD) implantation. While various markers of right ventricular function and right ventriculoarterial coupling have been proposed, their value in predicting in-hospital mortality remains unclear. This study aimed to investigate the prognostic significance of the right atrial pressure/pulmonary capillary wedge pressure (RAP/PCWP) ratio—a surrogate of RV–pulmonary artery (PA) coupling—for in-hospital mortality following LVAD implantation. **Methods**: This retrospective single-center study included 44 patients who underwent LVAD implantation. Preoperative clinical, echocardiographic, and invasive hemodynamic parameters were collected. The optimal RAP/PCWP ratio cut-off was determined using receiver operating characteristic (ROC) analysis. Predictors of in-hospital mortality were assessed using univariate and multivariate logistic regression. **Results**: Patients were stratified into high (≥0.47) and low (<0.47) RAP/PCWP ratio groups. In-hospital mortality was significantly higher in the high RAP/PCWP group (46% vs. 10%, *p* = 0.020). The optimal cut-off for the RAP/PCWP ratio was 0.47 (AUC: 0.829). In multivariate analysis, RAP/PCWP ratio (OR: 3.48 per 0.1 increase, *p* = 0.020) and INTERMACS 1–2 profile (OR: 39.19, *p* = 0.026) were independent predictors of in-hospital mortality. **Conclusions**: Preoperative RAP/PCWP ratio, as a surrogate of right ventriculoarterial coupling, independently predicts in-hospital mortality following LVAD implantation. Its incorporation into preoperative assessment may enhance risk stratification and guide clinical management in this high-risk population.

## 1. Introduction

Right ventricular failure (RVF) following left ventricular assist device (LVAD) implantation is associated with adverse outcomes—including higher mortality, increased bleeding, and longer hospitalization duration—and may complicate up to 40% of cases [1,2,3]. Patients who require right ventricular assist device (RVAD) support after LVAD implantation due to right ventricular dysfunction (RVD) have demonstrated significantly higher mortality in previous studies [1,2,3,4]. In the MOMENTUM cohort, 34% of patients developed RVF after LVAD (HeartMate 3) implantation, and RVAD placement following LVAD was found to be associated with a 21% to 42% 30-day mortality rate [4]. RVF was also identified as one of the leading causes of early death in the European Registry of Mechanical Circulatory Support (EUROMACS) [5]. Moreover, patients undergoing concurrent RVAD support at the time of LVAD implantation had worse 1-year survival (56% vs. 82%) compared with those receiving LVAD alone [1]. Despite the decreasing mortality rates due to the advances in device technology [6,7], RVF remains an established predictor of death in LVAD recipients [8,9,10].

Accurate preoperative prediction of RVF is critical in managing LVAD candidates. Various invasive and echocardiographic markers of RV function such as pulmonary artery pulsatile index (PAPi), right ventricle stroke work index (RVSWI), right atrial pressure/pulmonary capillary wedge pressure (RAP/PCWP) ratio, and tricuspid annular plane systolic excursion/pulmonary artery systolic pressure (TAPSE/PASP) ratio, have been evaluated to explore the predictors of RVD and death in LVAD recipients [11,12,13,14,15,16,17]. Among these, TAPSE/PASP, RAP/PCWP, and PAPi, which serve as surrogates of ventriculoarterial coupling have been associated with RVF and long-term mortality [11,12,13,14,15,16,17]. However, the utility of RV function and RV–pulmonary artery (PA) coupling measures for predicting in-hospital mortality after LVAD implantation remains undefined. This study aims to identify predictors of in-hospital mortality in LVAD recipients, with a particular focus on catheterization-derived parameters reflecting RV systolic function and RV–PA coupling.

## 2. Materials and Methods

This single-center study retrospectively analyzed prospectively gathered data on the patients who underwent LVAD implantation for advanced heart failure with reduced ejection fraction as destination therapy between 2 August 2019 and 5 May 2025. All patients were over 18 years of age, and none of the patients was excluded. Data on baseline clinical characteristics, laboratory values, regular medications, comorbidities, echocardiographic examination, and left and right heart catheterization were obtained from the hospital information system and patient files. All baseline clinical data, including comorbidities, laboratory values, and regular medications, were evaluated and recorded at the time of admission.

### 2.1. Echocardiography

Echocardiographic examinations were performed one day prior to LVAD implantation by experienced cardiac imaging specialists using a 3.5 MHz or M5S ultrasound probe and Vivid E95 ultrasound device (General Electric Vingmed Ultrasound, Milwaukee, WI, USA). Standard echocardiographic measurements included left ventricular ejection fraction (LVEF) calculated by the biplane Simpson’s method, left and right ventricular and left atrial dimensions, RV systolic function parameters such as TAPSE and peak systolic velocity of the tricuspid annulus by tissue Doppler imaging (RV-St). In addition, valvular pathologies and pulmonary artery pressures were also evaluated. Pulmonary artery systolic pressure (PASP) was calculated over tricuspid regurgitation using the Bernoulli equation: PASP = 4 × Vmax^2^ + estimated right atrial (RA) mean pressure. RA mean pressure was estimated based on the inferior vena cava diameter and its respiratory variation. All echocardiographic measurements were obtained in accordance with the guidelines of the European Association of Cardiovascular Imaging (EACVI) and the American Society of Echocardiography (ASE) [18].

### 2.2. Left and Right Heart Catheterization

All patients underwent left and right heart catheterization one day prior to LVAD implantation. Femoral arterial and venous accesses were obtained under ultrasound guidance. The mid-thoracic line of the supine patient was used as the zero level for pressure tracing. To record pressures in the left ventricle, aorta, pulmonary artery, right ventricle, and right atrium, 6F pigtail catheters were utilized. Swan–Ganz catheters were used to measure pulmonary capillary wedge pressure (PCWP). Cardiac output was calculated using the Fick principle. Cardiac index, stroke volume, and stroke volume index were derived by adjusting cardiac output for body surface area and heart rate.

The following formula was used to calculate the RV stroke work index:

RVSWI = (mean pulmonary artery pressure − mean right atrial pressure) × stroke volume index.

The surrogates of right ventriculoarterial coupling were as follows:

1. TAPSE/PASP ratio: Calculated using echocardiographic measurements, derived by dividing TAPSE (in cm) by the estimated PASP (in mmHg), which was obtained using the Bernoulli equation.

2. PAPi: Calculated as (systolic pulmonary artery pressure-diastolic pulmonary artery pressure)/RAP. All measurements were obtained from right heart catheterization.

3. RAP/PCWP: Defined as the ratio of right atrial mean pressure to pulmonary capillary wedge pressure, both measured invasively during right heart catheterization.

### 2.3. Ethics

Institutional ethics committee approval was obtained for this study. This study was conducted in accordance with the Declaration of Helsinki. Informed consent was obtained from all patients at admission and before all invasive procedures.

### 2.4. Statistical Analysis

The Kolmogorov–Smirnov test and visual histograms were used to evaluate the distribution of variables. Categorical variables are expressed as counts and percentages. Continuous variables with normal distributions are presented as mean ± standard deviation while non-normally distributed variables are presented as median and interquartile range. Group comparisons based on the RAP/PCWP ratio cut-off value were performed using the Chi-square test for categorical variables, and the Mann–Whitney U test for continuous variables. The optimal cut-off value for the RAP/PCWP ratio in predicting in-hospital mortality was determined using receiver operating characteristic (ROC) curve analysis and Youden’s index. We compared the discriminative ability of the RAP/PCWP ratio, PAPi, and TAPSE/PASP ratio for in-hospital mortality using pairwise area under the curve (AUC) comparisons. AUCs were estimated with the nonparametric method, and differences in AUCs were assessed by bootstrap (1000 resamples) to derive mean ΔAUC, 95% confidence intervals (percentile method), and two-sided *p*-values.

The primary outcome of this study was in-hospital mortality. Potential predictors—including baseline clinical characteristics, laboratory values, imaging findings, comorbidities, medications, echocardiographic parameters, and cardiac catheterization measurements—were first evaluated using univariate logistic regression analysis. Variables that were significant in univariate analysis, along with age, were subsequently included in multivariate logistic regression. The results of the multivariate model were visualized using a forest plot. As noted, the low number of events and small sample size increase the risk of overfitting and unstable estimates. To address this concern, we applied an L_2_-penalized logistic regression model and conducted 1000-resample bootstrap validation.

To evaluate whether RAP/PCWP adds independent prognostic information beyond a binary INTERMACS profile (1–2 vs. 3–7), we performed the following analyses:

1. Model specification

Model A: logistic regression for in-hospital mortality using INTERMACS profile as a binary predictor (1–2 vs. 3–7).

Model B: as Model A, with the addition of the RAP/PCWP ratio expressed per 0.1-unit increment.

2. Likelihood-ratio test (LRT)

We compared Model B against Model A using a χ^2^ test on twice the difference in log-likelihoods, with 1 degree of freedom.

3. Continuous net reclassification improvement (NRI)

We obtained 95% confidence intervals for the NRI via nonparametric bootstrap (1000 resamples), reporting the 2.5th and 97.5th percentiles.

A two-tailed *p*-value < 0.05 was considered statistically significant. All statistical analyses were conducted using Python version 3.11.

## 3. Results

The study population consisted of 44 patients. The mean age was 61.8 ± 10.3, and 36 patients (81.8%) were male. Then, patients were stratified into two groups based on the optimal RAP/PCWP ratio cut-off. The high RAP/PCWP group (≥0.47) included 13 patients, while the low RAP/PCWP ratio (<0.47) group included 31 patients. There were no significant differences in mean age (62.1 ± 11.1 vs. 61.7 ± 10.1 years, *p* = 0.908), body mass index (27.3 ± 4.5 vs. 30.0 ± 5.7 kg/m^2^, *p* = 0.136), or sex distribution (84.6% vs. 80.6% male, *p* = 1.000) between the groups (Table 1).

The prevalence of pre-existing comorbidities including hypertension, diabetes mellitus, chronic kidney disease, cerebrovascular accident, atrial fibrillation, and coronary artery disease, as well as INTERMACS profiles, were comparable between groups. Likewise, the use of cardiovascular medications such as angiotensin-converting enzyme inhibitors (ACEi)/angiotensin receptor blockers (ARBs), beta-blockers, angiotensin receptor/neprilysin inhibitor (ARNI), sodium-glucose co-transporter 2 inhibitors (SGLT-2i), mineralocorticoid receptor antagonists (MRAs), and loop diuretics did not differ between groups (Table 1). Laboratory values including hemoglobin, platelet count, C-reactive protein, creatinine, urea, aspartate aminotransferase (AST), alanine aminotransferase (ALT), albumin, sodium, potassium, total bilirubin, hemoglobin A1c, total cholesterol, low-density-lipoprotein (LDL)-cholesterol, thyroid-stimulating hormone (TSH), and N-terminal pro-B-type natriuretic peptide (NT-proBNP) levels were also comparable between two groups (Table 1).

Echocardiographic parameters including LVEF, left atrial (LA) anteroposterior dimension, LV end-diastolic and end-systolic dimensions, TAPSE/PASP ratio, RV-St, and RV basal diameters were compared between high- and low-RAP/PCWP groups. No significant differences were observed for any of these parameters (Table 2).

Hemodynamic parameters obtained from left and right catheterization were compared between two RAP/PCWP groups. Cardiac output (4.0 ± 1.0 L/dk vs. 3.9 ± 0.9 L/dk, *p* = 0.830), cardiac index (2.0 ± 0.5 L/dk/m^2^ vs. 2.1 ± 0.3 L/dk/m^2^, *p* = 0.603), stroke volume (50.9 ± 14.7 mL/beat vs. 53.6 ± 17.9 mL/beat), and stroke volume index (26.1 ± 7.9 mL/beat/m^2^ vs. 27.9 ± 7.5 mL/beat/m^2^, *p* = 0.559) were similar between the groups. No significant differences were observed in aortic and pulmonary pressures or pulmonary vascular resistance. RVSWI, an indicator of RV systolic function, was also comparable between the two groups (597.3 ± 331.0 mmHg·mL/m^2^ vs. 475.0 ± 342.5 mmHg·mL/m^2^, *p* = 0.341). However, PAPi, another surrogate marker of right ventriculoarterial coupling, was significantly lower in the high RAP/PCWP group (1.7 ± 0.9 vs. 3.5 ± 1.2, *p* < 0.001) (Table 2).

The incidence of arrhythmic events, including ventricular tachycardia and ventricular fibrillation, during hospitalization did not differ between the two groups (23% vs. 6%, *p* = 0.287), while the high RAP/PCWP group had a significantly higher rate of in-hospital mortality compared to the low RAP/PCWP group (46% vs. 10%, *p* = 0.02) (Table 2).

**Figure 1:**
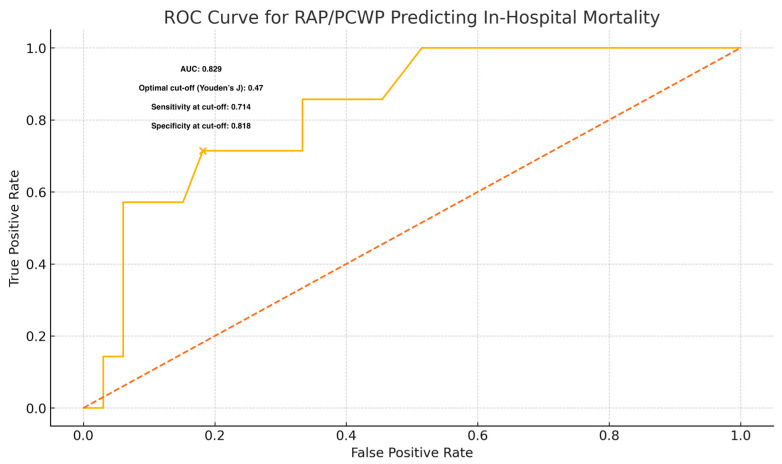
Receiver operating characteristic (ROC) curve analysis to determine the optimal cut-off value for the RAP/PCWP ratio in predicting in-hospital mortality.

ROC curve analysis was conducted to determine the optimal cut-off value of the RAP/PCWP ratio for predicting in-hospital mortality. The analysis identified a cut-off value of 0.47 based on Youden’s index, yielding a sensitivity of 71.4% and a specificity of 81.8%. The AUC was 0.829, indicating good discriminative ability (Figure 1). In predicting in-hospital mortality, RAP/PCWP achieved an AUC of 0.829, significantly higher than that of PAPI (AUC 0.307; mean ΔAUC 0.523; 95% CI 0.192–0.823; *p* = 0.008) and TAPSE/PASP (AUC 0.299; mean ΔAUC 0.533; 95% CI 0.224–0.807; *p* = 0.002). There was no significant difference between PAPI and TAPSE/PASP (ΔAUC 0.010; 95% CI −0.215–0.250; *p* = 0.972), confirming that RAP/PCWP provides superior discrimination among coupling indices (Table 3).

Variables including baseline clinical characteristics, LVAD type (HeartMate 3 vs. HeartMate 2), INTERMACS profile (1–2 vs. 3–7), pre-existing comorbidities, regular medications, laboratory values, echocardiographic parameters, the presence of arrhythmic events during hospitalization, and cardiac catheterization measurements were analyzed using univariate logistic regression. In this analysis, INTERMACS 1–2 profile (OR: 33.00, *p* < 0.001), albumin level (OR: 0.21, *p* = 0.022), and the RAP/PCWP ratio (OR: 1.97 per 0.1 increase, *p*= 0.017) were identified as significant predictors of in-hospital mortality (Table 4).

**Figure 2:**
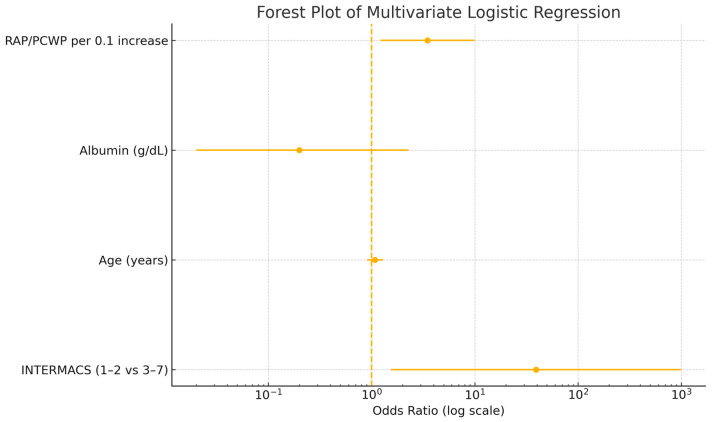
Forest plot of the multivariate logistic regression analysis for the prediction of in-hospital mortality.

These variables, along with age, were subsequently included in a multivariate logistic regression model. INTERMACS 1–2 profile (OR: 39.19, *p* = 0.026) and RAP/PCWP ratio (OR: 3.48 per 0.1 increase, *p* = 0.020) remained as independent predictors of in-hospital mortality (Table 5). The results of the multivariate analysis were visualized in a forest plot (Figure 2).

As noted, with only nine in-hospital deaths and four candidate predictors, the commonly accepted rule of at least 10 events per variable is not satisfied. This raises concerns about potential overfitting and the instability of parameter estimates in traditional multivariate logistic regression. To address this issue, we applied an L_2_-penalized logistic regression model, followed by 1000-resample bootstrap validation (Table 6). This approach confirmed the independent associations of the RAP/PCWP ratio and INTERMACS profile with in-hospital mortality, while offering more robust estimates than conventional logistic regression. The apparent odds ratios for INTERMACS profile (1–2 vs. 3–7) and RAP/PCWP (per 0.1 increase) were 3.88 and 2.19, respectively. As indicators of model stability, the shrinkage factor was 1.01—suggesting minimal overfitting—while the apparent and optimism-corrected C-statistics were 0.957 and 0.922, respectively.

Adding RAP/PCWP to the binary INTERMACS model (1–2 vs. 3–7) significantly improved prognostic performance:

Likelihood-ratio test: Δχ^2^(1) = 8.13, *p* = 0.004, indicating Model B fits the data better than INTERMACS alone.

Continuous NRI: 1.19 (95% CI: 0.55–2.00), demonstrating substantial net reclassification of both events and non-events when RAP/PCWP is added.

These findings confirm that RAP/PCWP provides significant incremental prognostic value over a simple binary INTERMACS classification.

## 4. Discussion

RVF following LVAD implantation is defined as the inability of the RV to provide adequate cardiac output to support both the left ventricle and LVAD device. After LVAD implantation, the increase in coronary perfusion and the reduction in LV end-diastolic pressure (LVEDP) provide improved myocardial contractility. However, acute LV unloading can lead to a leftward septal shift, impairing RV contractility and increasing its sensitivity to afterload changes [1,19]. Additionally, the loss of pericardial constraint after surgical pericardiotomy, combined with increased cardiac output, may result in increased venous return and RV preload. This hemodynamic burden may exacerbate RVF, particularly in a previously maladapted or compromised RV [1,19].

The RAP/PCWP ratio provides an estimate of right-to-left heart filling pressure balance and has been proposed as a surrogate of right ventricular–pulmonary arterial coupling. A high RAP/PCWP ratio reflects elevated right atrial pressure relative to left-sided filling pressure, suggesting impaired right ventricular function or disproportionate RV afterload. In the context of advanced heart failure, a coupled RV can accommodate increased PCWP without a significant rise in RAP, maintaining forward flow. However, when RV contractile reserve is exhausted, RAP rises disproportionately—resulting in a high RAP/PCWP ratio. Following LVAD implantation, left ventricular unloading reduces PCWP, which should theoretically lower RAP if the RV is able to compensate. However, in patients with RV–PA uncoupling, the RV fails to accommodate this shift in preload and afterload, leading to persistent or rising RAP despite falling PCWP. This mismatch reflects a maladaptive RV response to changes in loading conditions and contributes to postoperative RV failure and adverse outcomes. Thus, a high preoperative RAP/PCWP ratio may identify patients whose right ventricle is already uncoupled and less likely to tolerate the hemodynamic shifts following LVAD initiation [8,11,13,19,20,21].

The presence of RV dysfunction in addition to LV failure is associated with increased mortality and adverse outcomes in patients with advanced heart failure [20,21,22]. Elevated PCWP increases RV afterload, and when RV fails to compensate, clinical signs of RV failure may become evident. Moreover, the absence of effective RV–PA coupling indicates RV dysfunction superimposed on LV failure, and is associated with worse prognosis, higher mortality, and increased adverse outcomes in patients with advanced heart failure [20,21,22]. Isolated markers of RV systolic function such as TAPSE, RV-St, and RVSWI are highly afterload-dependent and may lead to the misinterpretation of RV function. In contrast, RV–PA coupling surrogates, such as the RA/PCWP ratio, PAPi, and TAPSE/PASP ratio, provide more accurate assessments of RV function by incorporating afterload dynamics. Therefore, these ventriculoarterial coupling measures are considered to have greater prognostic significance in patients with advanced heart failure [20,21,22].

The predictive value of ventriculoarterial coupling markers in LVAD recipients has been previously investigated. Pre-implant RV uncoupling appears to be associated with an increased risk of post-implant RV dysfunction and higher long-term mortality [5,6,8,11,12,13,14,15,16,17]. Mehra et al. showed that higher pre-implant RAP/PCWP ratio values were associated with increased 1-year and 2-year mortality [6]. Similarly, data from the ASSIST-ICD registry indicated that the RAP/PCWP ratio was a predictor of long-term all-cause mortality in LVAD recipients, whereas other coupling markers such as the TAPSE/PASP ratio and PAPi were not [11]. In addition, Ruiz-Cano et al. demonstrated that the central venous pressure (CVP)/PCWP ratio predicts early RVF failure after LVAD implantation [15]. A decreasing RAP/PCWP ratio after LVAD support suggests an improvement in RV function [23]. Lower PAPi values, as another marker of RV–PA uncoupling, were also associated with an increased risk of RVF and long-term mortality after LVAD implantation [12,13,14,16]. The TAPSE/PASP ratio has similarly been shown to correlate with RVD development in this population [17]. In these studies, isolated RV systolic function parameters such as RV-SWI and TAPSE—which do not account for changes in afterload—could not demonstrate prognostic value in predicting RVF or long-term mortality in LVAD recipients [5,6,8,11,12,13,14,15,16,17].

Although the development of RVF remains among the significant causes of death in LVAD recipients [2,5,8,24], and the prognostic impact of right ventriculoarterial coupling surrogates on RVF development and long-term outcomes following LVAD implantation is well-established [5,6,8,11,12,13,14,15,16,17], their utility in predicting in-hospital mortality in this patient subset is not yet defined. In previously developed risk models for in-hospital mortality in LVAD patients, echocardiographic and hemodynamic indicators of RV–PA coupling have been underrepresented. Instead, clinical variables—such as INTERMACS profile, renal failure, mechanical ventilation, age, and heart rate—and laboratory parameters including albumin, hemoglobin, lactate, creatinine, international normalized ratio (INR), and bilirubin have been more prominently featured as prognostic factors [5,8,22,25]. Pettinari et al. evaluated three risk scores including Fitzpatrick’s score, Drakos’ score, and Matthews’ score, with the following components:

- Fitzpatrick’s score = 18 (cardiac index, L/min) + 18 (RV stroke work index, mmHg L/m^2^) + 17 (creatinine, mg/dL) + 16 (previous cardiac surgery) + 16 (RV dysfunction) + 13 (systolic blood pressure, mmHg).
- Drakos’ score = 3.5 (destination therapy) + 4 (intra-aortic balloon) + 4 (pulmonary vascular resistance: one if PVR < 1.7 Wood units, two if 1.8–2.7 Wood units, three if 2.8–4.2 Wood units, and four if >4.3 Wood units) + 2.5 (inotrope dependency) + 2 (obesity) + 2.5 (angiotensin converting enzyme inhibitor and/or angiotensin II receptor blocker) + 2 (β-blocker).
- Matthews’ score = 4 (vasopressor requirement) + 2 (if AST ≥80 IU/L) + 2.5 (if bilirubin ≥2.0 mg/dL) + 3 (creatinine ≥2.3 mg/dL or renal replacement therapy).

These risk scores include only a limited set of hemodynamic variables—namely cardiac index, right ventricular stroke work index (RVSWI), and pulmonary vascular resistance—while omitting more comprehensive surrogates of right ventriculoarterial coupling. Notably, measures of left ventricular (LV) function become clinically less relevant after LVAD implantation, and afterload-dependent parameters such as RVSWI may lead to misinterpretation when assessing intrinsic RV function. Consequently, none of these scores have demonstrated reliable predictive value for RVF following LVAD implantation.

In contrast, parameters reflecting RV–pulmonary artery (PA) coupling may offer superior prognostic insight by accounting for the RV’s ability to adapt to changes in afterload. In this study, we demonstrated that the RAP/PCWP ratio—a well-established marker of ventriculoarterial coupling and a known predictor of long-term mortality and RVF—also retains its prognostic value in predicting in-hospital mortality among LVAD recipients.

In our study, the RAP/PCWP ratio emerged as an independent predictor of in-hospital mortality among the markers of RV–pulmonary artery (PA) coupling. While the TAPSE/PASP ratio has previously demonstrated concordance with invasive measures of ventriculoarterial coupling, it has several important limitations. TAPSE primarily reflects the longitudinal motion of the basal free wall of the right ventricle, but does not account for other components of RV function, such as the radial contraction of the free wall or septal contribution. Additionally, an accurate estimation of PASP by echocardiography is not feasible in all patients—particularly when tricuspid regurgitation is inadequate or tricuspid leaflet coaptation is absent. Furthermore, the TAPSE/PASP ratio is subject to angle dependency and operator variability, which may introduce subjectivity and reduce its reproducibility. Stąpór et al. [26] and Sert al. [17] demonstrated the prognostic value of echocardiographic measures of right ventriculoarterial coupling such as RV free wall strain/PASP and TAPSE/PASP ratio in predicting long-term mortality or late RVF after LVAD implantation. However, these parameters did not predict early RVF or in-hospital mortality. This limitation underscores the potential advantage of invasive hemodynamic markers, which may offer more accurate and reproducible assessments of RV–PA coupling. In this study, although a trend toward increased in-hospital mortality was observed with decreasing PAPi values (OR: 0.51, 95% CI: 0.23–1.13, *p* = 0.09), this association did not reach statistical significance. While PAPi is a well-established marker of right ventriculoarterial coupling and has been shown to predict RV dysfunction and long-term mortality in LVAD recipients, the lack of significance in our analysis may be attributed to the limited sample size, which could reduce statistical power to detect its prognostic effect for in-hospital mortality. In contrast, the RAP/PCWP ratio—a surrogate of RV–PA coupling—demonstrated a stronger and statistically significant association with in-hospital mortality in the multivariate analysis. Moreover, RAP/PCWP ratio showed superior discriminative power relative to other RV–PA coupling indices for predicting in-hospital mortality. Importantly, this association persisted even after adjusting for INTERMACS 1–2 profile, a well-established predictor of early mortality in LVAD candidates, underscoring the independent prognostic value of the RAP/PCWP ratio in this setting. This association remained significant in the L_2_-penalized logistic regression analysis, which was followed by 1000-resample bootstrap validation to minimize bias and assess potential overfitting. Although the Youden index-derived RAP/PCWP threshold of 0.47 yielded high specificity (82%), its sensitivity was only 71%, implying that nearly 30% of in-hospital deaths would be missed if RAP/PCWP ratios were applied in isolation. This underscores that RAP/PCWP should be used as a component of risk factors rather than as a standalone screening tool. While RAP/PCWP alone (threshold 0.47) shows only moderate sensitivity, its inclusion alongside categorical INTERMACS profile (1–2 vs. 3–7) significantly improves model discrimination (Δχ^2^ = 8.13, *p* = 0.004) and yields a continuous NRI of 1.19 (95% CI 0.55–2.00). This confirms that RAP/PCWP adds substantial prognostic information beyond clinical profile alone, supporting an integrated risk-stratification approach.

Because hemodynamic measurements were obtained approximately one day prior to LVAD implantation, it is possible that preoperative interventions—such as diuretics, vasodilators, or inotropes—may have influenced RAP/PCWP values. As such, our findings reflect the prognostic value of RAP/PCWP under real-world conditions rather than representing unadjusted baseline physiology. The potential for confounding due to hemodynamic optimization should be considered when interpreting the association with in-hospital mortality.

Our study population consisted of patients who underwent LVAD implantation as destination therapy, and therefore included relatively older individuals with multiple comorbidities. Notably, 43.2% of patients were classified as INTERMACS 1–3, indicating a higher risk profile with respect to in-hospital mortality. For these reasons, our observed in-hospital mortality rate (20.4%) falls at the upper end of the reported range. Our study demonstrated the utility of the RAP/PCWP ratio as a predictor of in-hospital mortality in a high-risk patient group. However, larger studies are warranted to evaluate its applicability in lower-risk populations. The results of this study require confirmation in larger, preferably multicenter cohorts.

## 5. Conclusions

Preoperative RAP/PCWP ratio, as a surrogate of right ventriculoarterial coupling, independently predicts in-hospital mortality following LVAD implantation. Careful assessment of this parameter in the preoperative period may enhance risk stratification and guide clinical management in this high-risk population.

## Figures and Tables

**Table 1:** Comparison of baseline clinical characteristics, medications, and laboratory parameters based on RAP/PCWP ratio.

Variables	RAP/PCWP < 0.47 (*n* = 31)	RAP/PCWP ≥ 0.47 (*n* = 13)	*p*-Value
***Baseline Clinical Characteristics***			
Age (years)	61.7 ± 10.1	62.1 ± 11.1	0.908
Male Sex	25 (80.6%)	11 (84.6%)	1.00
Body Mass Index (kg/m^2^)	30.0 ± 5.7	27.3 ± 4.5	0.136
INTERMACS Profile			
INTERMACS 1	2 (6.4%)	2 (15.4%)	0.393
INTERMACS 2	2 (6.4%)	2 (15.4%)	
INTERMACS 3	8 (25.8%)	3 (23.1%)	
INTERMACS 4	14 (45.2%)	3 (23.1%)	
INTERMACS 5	3 (9.7%)	2 (15.4%)	
INTERMACS 6	2 (6.4%)	0 (0%)	
INTERMACS 7	0 (0%)	1 (7.6%)	
Presence of ICD	25 (80.6%)	11 (84.6%)	1.00
Hypertension	25 (80.6%)	11 (84.6%)	1.00
Diabetes Mellitus	18 (58.1%)	4 (30.7%)	0.186
Chronic Kidney Disease	17 (54.8%)	6 (46.1%)	0.845
Cerebrovascular Accident	1 (3.2%)	1 (7.7%)	1.00
Atrial Fibrillation	13 (41.9%)	3 (23.1%)	0.399
Coronary Artery Disease	22 (70.9%)	10 (76.9%)	0.973
Previous PCI	19 (61.3%)	5 (38.4%)	0.291
Previous CABG	3 (9.7%)	5 (38.4%)	0.067
LVAD Type			0.721
HeartMate 3	22 (70.9%)	8 (61.6%)	
HeartMate 2	9 (29.1%)	5 (38.4%)	
***Regular Medications***			
Beta-Blocker Usage	27 (87.1%)	12 (92.3%)	1.00
ACEi/ARB Usage	6 (19.4%)	5 (38.4%)	0.340
ARNI Usage	12 (38.7%)	3 (23.1%)	0.516
SGLT-2i Usage	13 (41.9%)	4 (30.7%)	0.723
MRA Usage	14 (45.2%)	6 (46.1%)	1.00
Loop Diuretic Usage	25 (80.6%)	11 (84.6%)	1.00
***Laboratory Parameters***			
Hemoglobin (g/dL)	11.3 ± 2.3	11.4 ± 2.0	0.930
WBC (cell count/L)	(10.1 ± 3.5) × 10^3^	(7.9 ± 2.5) × 10^3^	0.045
Platelets (cell count/mcL)	(204.9 ± 79.8) × 10^3^	(202.3 ± 47.2) × 10^3^	0.914
CRP (mg/dL)	42.4 ± 40.7	30.9 ± 39.6	0.395
Creatinine (mg/dL)	1.8 ± 1.2	1.6 ± 1.3	0.579
Urea (mg/dL)	76.0 ± 45.4	92.5 ± 100.7	0.468
AST (IU/L)	139.7 ± 337.6	41.5 ± 26.6	0.324
ALT (IU/L)	123.5 ± 278.1	25.4 ± 16.0	0.253
Albumin (g/dL)	3.7 ± 0.7	3.6 ± 0.5	0.751
Sodium (mEq/L)	135.3 ± 3.7	135.3 ± 5.5	0.962
Potassium (mEq/L)	4.2 ± 0.5	4.1 ± 0.5	0.614
Total Bilirubin (mg/dL)	0.9 ± 0.7	0.9 ± 0.3	0.853
LDH (units/L)	395.2 ± 528.6	230.2 ± 82.5	0.551
HbA1c (%)	7.1 ± 1.6	6.5 ± 0.7	0.641
Total Cholesterol (mg/dL)	153.9 ± 43.7	141.8 ± 42.0	0.402
LDL Cholesterol (mg/dL)	98.9 ± 28.1	81.0 ± 37.1	0.115
TSH (mIU/L)	2.5 ± 2.0	4.6 ± 4.6	0.084
NT-proBNP (pg/mL)	5199.5 (3720.2–10,084.5)	6323 (5648–8233)	0.713

Abbreviations: RAP: right atrial mean pressure, PCWP: pulmonary capillary wedge pressure, INTERMACS: Interagency Registry for Mechanically Assisted Circulatory Support, ICD: implantable cardioverter defibrillator, PCI: percutaneous coronary intervention, CABG: coronary artery bypass graft surgery, LVAD: left ventricular assist device, ACEi: angiotensin converting enzyme inhibitors, ARB: angiotensin II receptor blocker, ARNI: angiotensin receptor/neprilysin inhibitor, SGLT-2i: sodium-glucose cotransporter-2 inhibitors, MRA: mineralocorticoid receptor antagonist, WBC: white blood cell, NT-proBNP: N-terminal prohormone of brain natriuretic peptide, AST: aspartate aminotransferase, ALT: alanine aminotransferase, CRP: C-reactive protein, LDL: low-density lipoprotein, LDH: lactate dehydrogenase, TSH: thyroid-stimulating hormone, and HbA1c: hemoglobin A1c.

**Table 2:** Comparison of echocardiographic parameters, cardiac catheterization measurements, and outcomes based on RAP/PCWP ratio.

Variables	RAP/PCWP < 0.47 (*n* = 31)	RAP/PCWP ≥ 0.47 (*n* = 13)	*p*-Value
***Echocardiographic Parameters***			
LVEF (%)	19.2 ± 3.2	19.2 ± 4.2	0.982
Left Atrium AP Diameter (cm)	5.1 ± 0.3	5.0 ± 0.2	0.945
LV End-diastolic Diameter (cm)	7.3 ± 0.6	7.1 ± 1.0	0.555
LV End-systolic Diameter (cm)	6.3 ± 0.6	6.5 ± 0.7	0.652
TAPSE/PASP (mm/mmHg)	0.3 ± 0.1	0.3 ± 0.1	0.984
RV-St (cm/sn)	9.6 ± 1.3	9.5 ± 1.3	0.906
RV Basal Diameter (cm)	4.0 ± 0.5	3.9 ± 0.6	0.487
MR			0.144
Grade 0	0 (0%)	1 (7.6%)	
Grade 1	0 (0%)	1 (7.6%)	
Grade 2	5 (16.1%)	4 (30.8%)	
Grade 3	14 (45.1%)	4 (30.8%)	
Grade 4	12 (38.7%)	3 (23.1%)	
AR			0.833
Grade 0	15 (48.4%)	5 (38.4%)	
Grade 1	14 (45.2%)	7 (53.9%)	
Grade 2	2 (6.4%)	1 (7.6%)	
Grade 3	0 (0%)	0 (0%)	
Grade 4	0 (0%)	0 (0%)	
TR			0.379
Grade 0	0 (0%)	0 (0%)	
Grade 1	10 (32.2%)	5 (38.4%)	
Grade 2	16 (51.6%)	4 (30.7%)	
Grade 3	4 (12.9%)	2 (15.4%)	
Grade 4	1 (3.2%)	2 (15.4%)	
***Left and Right Heart Catheterization Measurements***			
Cardiac Output (L/min)	4.0 ± 1.0	3.9 ± 0.9	0.830
Cardiac Index (L/min/m^2^)	2.0 ± 0.5	2.1 ± 0.3	0.603
Stroke Volume (mL/beat)	50.9 ± 14.7	53.6 ± 17.9	0.648
Stroke Volume Index (mL/beat/m^2^)	26.1 ± 7.9	27.9 ± 7.5	0.559
PCWP (mmHg)	25.7 ± 8.4	24.8 ± 7.4	0.773
PASP (mmHg)	48.5 ± 16.8	48.9 ± 21.8	0.953
PAPM (mmHg)	31.1 ± 11.1	31.6 ± 10.8	0.907
PAPD (mmHg)	21.6 ± 8.7	21.4 ± 5.2	0.956
PVR (Wood units)	1.9 ± 1.9	2.1 ± 1.8	0.767
Aortic Systolic Pressure (mmHg)	98.2 ± 12.9	109.3 ± 31.1	0.121
Aortic Mean Pressure (mmHg)	73.1 ± 6.8	79.9 ± 18.3	0.099
Aortic Diastolic Pressure (mmHg)	56.0 ± 14.6	64.2 ± 13.5	0.141
RAP (mmHg)	8.3 ± 3.8	15.6 ± 5.0	**<0.001**
RVSWI (mmHg·mL/m^2^)	597.3 ± 331.0	475.0 ± 342.5	0.341
PAPi	3.5 ± 1.2	1.7 ± 0.9	**<0.001**
***Outcomes***			
Arrhythmic Event During Hospitalization (VT/VF)	2 (6.4%)	3 (23.1%)	0.287
In-hospital Mortality	3 (9.7%)	6 (46.1%)	**0.020**

Abbreviations: RAP: right atrial mean pressure, PCWP: pulmonary capillary wedge pressure, LVEF: left ventricular ejection fraction, AP: anteroposterior, TAPSE: tricuspid annular plane systolic excursion, PASP: pulmonary artery systolic pressure, RV-St: peak systolic velocity of tricuspid annulus by tissue Doppler imaging, MR: mitral regurgitation, AR: aortic regurgitation, TR: tricuspid regurgitation, PAPM: pulmonary artery mean pressure, PAPD: pulmonary artery diastolic pressure, PVR: pulmonary vascular resistance, RVSWI: right ventricular stroke work index, PAPi: pulmonary artery pulsatility index, VT: ventricular tachycardia, and VF: ventricular fibrillation.

**Table 3:** Pairwise comparison of ROC AUCs for RAP/PCWP, PAPI, and TAPSE/PASP in predicting in-hospital mortality.

Comparison	AUC 1	AUC 2	AUC 3	Mean ΔAUC	95% CI ΔAUC	*p*-Value
RAP/PCWP ratio vs. PAPi	0.829	0.307	-	0.523	0.192–0.823	0.008
RAP/PCWP vs. TAPSE/PASP ratio	0.829	-	0.299	0.533	0.224–0.807	0.002
PAPi vs. TAPSE/PASP ratio	-	0.307	0.299	0.010	−0.215–0.250	0.972

Abbreviations: RAP: right atrial mean pressure, PCWP: pulmonary capillary wedge pressure, TAPSE: tricuspid annular plane systolic excursion, PASP: pulmonary artery systolic pressure, PAPi: pulmonary artery pulsatility index, ROC: receiver operating characteristic, and AUC: area under curve.

**Table 4:** Univariate logistic regression analysis for the prediction of in-hospital mortality.

Variables	Odds Ratio	95% Confidence Interval	*p*-Value
***Clinical Characteristics***			
INTERMACS profile (1–2 vs. 3–7)	33.00	4.51–241.28	**<0.001**
Age (per 1 year)	1.07	0.99–1.17	0.097
Sex (female)	0.50	0.05–4.69	0.544
Body mass index (per 1 kg/m^2^)	0.99	0.86–1.14	0.878
LVAD type (HeartMate 3 vs. HeartMate 2)	0.50	0.11–2.253	0.367
VT/VF during hospitalization	3.05	0.43–21.79	0.267
Pre-existing hypertension	2.00	0.21–18.74	0.544
Pre-existing diabetes mellitus	0.76	0.17–3.29	0.709
Pre-existing atrial fibrillation	1.53	0.35–6.79	0.574
Previous CAD	1.40	0.25–7.93	0.704
Previous PCI	1.05	0.24–4.59	0.946
Previous CABG	1.38	0.23–8.36	0.725
Presence of ICD	2.00	0.21–18.74	0.544
***Regular Medications***			
ACEi/ARB usage	1.69	0.34–8.31	0.520
Beta-blocker usage	1.03	0.10–10.56	0.979
SGLT-2i usage	0.15	0.02–1.32	0.087
MRA usage	0.53	0.11–2.46	0.417
***Laboratory Parameters***			
Hemoglobin (per 1 mg/dL)	0.84	0.58–1.20	0.330
CRP (per 1 mg/dL)	1.01	0.99–1.02	0.430
Creatinine (per 1 mg/dL)	1.72	0.96–3.07	0.066
AST (per 1 IU/L)	1.00	0.99–1.00	0.563
Albumin (per 1 g/dL)	0.21	0.06–0.80	**0.022**
Sodium (per 1 mEq/L)	0.83	0.68–1.02	0.081
Total bilirubin (per 1 mg/dL)	1.33	0.38–4.65	0.659
Total cholesterol (per 1 mg/dL)	1.00	0.98–1.01	0.625
LDL cholesterol (per 1 mg/dL)	0.99	0.96–1.01	0.366
NT-proBNP (logarithmic)	1.16	0.41–3.28	0.784
***Echocardiographic Parameters***			
LVEF (per 1%)	1.03	0.83–1.28	0.798
LVEDD (per 1 cm)	2.03	0.55–7.45	0.286
TAPSE/PASP ratio (per 0.1 mm/mmHg)	0.82	0.40–1.67	0.585
RV-St (per 1 cm/sn)	1.07	0.42–2.75	0.887
RV basal diameter (per 1 cm)	1.00	0.19–5.20	0.996
***Cardiac Catheterization Measurements***			
Cardiac output (per 1 L/min)	1.19	0.54–2.64	0.667
Cardiac index (per 1 L/min/m^2^)	2.25	0.45–11.31	0.326
Stroke volume (per 1 mL/beat)	0.99	0.94–1.05	0.792
Stroke volume index (per 1 mL/beat/m^2^)	1.08	0.97–1.20	0.141
PCWP (per 1 mmHg)	1.03	0.93–1.15	0.545
PASP (per 1 mmHg)	1.02	0.97–1.06	0.480
PAPM (per 1 mmHg)	1.02	0.95–1.10	0.545
PAPD (per 1 mmHg)	1.01	0.91–1.12	0.833
PVR (per 1 Woods unit)	0.79	0.46–1.38	0.411
RAP/PCWP (per 0.1 increase)	1.97	1.12–3.47	**0.017**
RVSWI (per 100 mmHg·mL/m^2^)	1.05	0.83–1.34	0.672
PAPi (per 1 increase)	0.51	0.23–1.13	0.098

Abbreviations: RAP: right atrial mean pressure, PCWP: pulmonary capillary wedge pressure, INTERMACS: Interagency Registry for Mechanically Assisted Circulatory Support, ICD: implantable cardioverter defibrillator, PCI: percutaneous coronary intervention, CABG: coronary artery bypass graft surgery, CAD: coronary artery disease, LVAD: left ventricular assist device, ACEi: angiotensin converting enzyme inhibitors, ARB: angiotensin II receptor blocker, SGLT-2i: sodium-glucose cotransporter-2 inhibitors, MRA: mineralocorticoid receptor antagonist, NT-proBNP: N-terminal prohormone of brain natriuretic peptide, AST: aspartate aminotransferase, CRP: C-reactive protein, LDL: low-density lipoprotein, LVEF: left ventricular ejection fraction, LVEDD: left ventricular end-diastolic diameter, TAPSE: tricuspid annular plane systolic excursion, PASP: pulmonary artery systolic pressure, RV-St: peak systolic velocity of tricuspid annulus by tissue Doppler imaging, PAPM: pulmonary artery mean pressure, PAPD: pulmonary artery diastolic pressure, PVR: pulmonary vascular resistance, RVSWI: right ventricular stroke work index, PAPi: pulmonary artery pulsatility index, VT: ventricular tachycardia, and VF: ventricular fibrillation.

**Table 5:** Multivariate logistic regression analysis for the prediction of in-hospital mortality.

Variable	Odds Ratio	95% Confidence Interval	*p*-Value
INTERMACS Profile (1–2 vs. 3–7)	39.19	1.54–996.64	0.026
Age (per 1 year)	1.08	0.91–1.29	0.353
Albumin (per 1 g/dL)	0.20	0.02–2.29	0.196
RAP/PCWP (per 0.1 increase)	3.48	1.22–9.94	0.020

Abbreviations: RAP: right atrial mean pressure, PCWP: pulmonary capillary wedge pressure, and INTERMACS: Interagency Registry for Mechanically Assisted Circulatory Support.

**Table 6:** L_2_-Penalized logistic regression and 1000-resample bootstrap validation for predicting in-hospital mortality.

Variable	Apparent Odds Ratio	Bootstrap Median Odds Ratio	95% Confidence Interval	Bootstrap-Derived *p*-Values
INTERMACS Profile (1–2 vs. 3–7)	3.88	3.62	1.37–6.99	0.03
Age (per 1 year)	1.07	1.08	0.97–1.19	0.16
Albumin (per 1 g/dL)	0.43	0.45	0.28–0.62	0.07
RAP/PCWP (per 0.1 increase)	2.19	2.27	1.39–4.46	<0.001

Abbreviations: RAP: right atrial mean pressure, PCWP: pulmonary capillary wedge pressure, and INTERMACS: Interagency Registry for Mechanically Assisted Circulatory Support.

## Data Availability

The datasets generated during and/or analyzed during the current study are not publicly available, but are available from the corresponding author on reasonable request.
